# Input-output relation and energy efficiency in the neuron with different spike threshold dynamics

**DOI:** 10.3389/fncom.2015.00062

**Published:** 2015-05-27

**Authors:** Guo-Sheng Yi, Jiang Wang, Kai-Ming Tsang, Xi-Le Wei, Bin Deng

**Affiliations:** ^1^School of Electrical Engineering and Automation, Tianjin UniversityTianjin, China; ^2^Department of Electrical Engineering, The Hong Kong Polytechnic UniversityHong Kong, China

**Keywords:** spike threshold dynamic, input-output relation, energy efficiency, biophysical connection, spike initiation

## Abstract

Neuron encodes and transmits information through generating sequences of output spikes, which is a high energy-consuming process. The spike is initiated when membrane depolarization reaches a threshold voltage. In many neurons, threshold is dynamic and depends on the rate of membrane depolarization (*dV*/*dt*) preceding a spike. Identifying the metabolic energy involved in neural coding and their relationship to threshold dynamic is critical to understanding neuronal function and evolution. Here, we use a modified Morris-Lecar model to investigate neuronal input-output property and energy efficiency associated with different spike threshold dynamics. We find that the neurons with dynamic threshold sensitive to *dV*/*dt* generate discontinuous frequency-current curve and type II phase response curve (PRC) through Hopf bifurcation, and weak noise could prohibit spiking when bifurcation just occurs. The threshold that is insensitive to *dV*/*dt*, instead, results in a continuous frequency-current curve, a type I PRC and a saddle-node on invariant circle bifurcation, and simultaneously weak noise cannot inhibit spiking. It is also shown that the bifurcation, frequency-current curve and PRC type associated with different threshold dynamics arise from the distinct subthreshold interactions of membrane currents. Further, we observe that the energy consumption of the neuron is related to its firing characteristics. The depolarization of spike threshold improves neuronal energy efficiency by reducing the overlap of Na^+^ and K^+^ currents during an action potential. The high energy efficiency is achieved at more depolarized spike threshold and high stimulus current. These results provide a fundamental biophysical connection that links spike threshold dynamics, input-output relation, energetics and spike initiation, which could contribute to uncover neural encoding mechanism.

## Introduction

Neurons, as the basic information-processing unit of the nervous system, can accurately represent and transmit various spatiotemporal patterns of sensory input in the form of sequences of output spikes (Koch, 1999; Dayan and Abbott, 2005; Klausberger and Somogyi, 2008). The generation and conduction of action potentials need to consume a lot of energy, which would have a great impact on neural codes and circuits (Niven and Laughlin, 2008; Alle et al., 2009; Sengupta et al., 2010, 2013, 2014; Moujahid et al., 2011). Characterizing energy efficiency associated with different input-output relations is an essential step toward capturing the full strategies used by the neuron to encode stimulus. Previous experimental and modeling studies (Koch, 1999; Dayan and Abbott, 2005; Klausberger and Somogyi, 2008; Niven and Laughlin, 2008; Prescott et al., 2008a; Alle et al., 2009; Carter and Bean, 2009; Sengupta et al., 2010, 2013, 2014) have reported that both of the input-output relation and energy efficiency of neurons depend not only on input spatiotemporal properties but also on neuronal intrinsic characteristics.

One basic intrinsic property for all spiking neurons is the spike threshold, which is a special membrane potential that distinguishes subthreshold responses from spikes (Izhikevich, 2005; Goldberg et al., 2008). The small depolarization of membrane potential below this special value is subthreshold and decays to resting potential, while large depolarization above this value is suprathreshold and results in an action potential (Izhikevich, 2005; Prescott et al., 2008a; Wester and Contreras, 2013). That is, a spike is initiated only when membrane depolarization reaches this threshold potential. *In vivo*, the spike threshold is dynamic, and varies with input properties as well as spiking history. Especially, it is inversely correlated with the preceding rate of membrane depolarization (i.e., *dV*/*dt*) prior to spike initiation (Azouz and Gray, 2000, 2003; Henze and Buzsáki, 2001; Ferragamo and Oertel, 2002; Escabí et al., 2005; Wilent and Contreras, 2005; Kuba et al., 2006; Goldberg et al., 2008; Priebe and Ferster, 2008; Cardin et al., 2010; Higgs and Spain, 2011; Platkiewicz and Brette, 2011; Wester and Contreras, 2013; Fontaine et al., 2014). A dynamic threshold plays a critically important role in spike generation, which would participate in and produce profound influences on neuronal input-output properties (Azouz and Gray, 2000, 2003; Henze and Buzsáki, 2001; Ferragamo and Oertel, 2002; Escabí et al., 2005; Wilent and Contreras, 2005; Kuba et al., 2006; Priebe and Ferster, 2008; Cardin et al., 2010; Platkiewicz and Brette, 2011). For instance, the neuron with a dynamic threshold is more capable of filtering out synaptic inputs (Higgs and Spain, 2011) and regulating its response sensitivity (Azouz and Gray, 2000, 2003; Ferragamo and Oertel, 2002; Wilent and Contreras, 2005; Cardin et al., 2010). Further, the dynamic threshold could also effectively enhance feature selectivity (Azouz and Gray, 2003; Escabí et al., 2005; Wilent and Contreras, 2005; Priebe and Ferster, 2008), contribute to coincidence detection and gain modulation (Azouz and Gray, 2000, 2003; Platkiewicz and Brette, 2011), as well as facilitate precise temporal coding (Kuba et al., 2006; Higgs and Spain, 2011).

The spike threshold dynamics could be modulated by the biophysical properties of intrinsic membrane currents (Hodgkin and Huxley, 1952; Azouz and Gray, 2000, 2003; Wilent and Contreras, 2005; Guan et al., 2007; Goldberg et al., 2008; Higgs and Spain, 2011; Platkiewicz and Brette, 2011; Wester and Contreras, 2013; Fontaine et al., 2014). Two especially relevant biophysical mechanisms are Na^+^ inactivation and K^+^ activation, which are originally recognized by Hodgkin and Huxley (1952). Because Na^+^ inactivation specifically affects spike initiation (Platkiewicz and Brette, 2011), it is usually regarded as the fundamental mechanism of regulating threshold (Azouz and Gray, 2000, 2003; Henze and Buzsáki, 2001; Wilent and Contreras, 2005; Platkiewicz and Brette, 2011; Wester and Contreras, 2013; Fontaine et al., 2014). Recently, more and more studies find that the outward K^+^ channels, especially those activated at the subthreshold potentials, could also powerfully regulate spike threshold (Storm, 1988; Bekkers and Delaney, 2001; Dodson et al., 2002; Guan et al., 2007; Goldberg et al., 2008; Higgs and Spain, 2011; Wester and Contreras, 2013). Blocking them (Storm, 1988; Bekkers and Delaney, 2001; Dodson et al., 2002; Guan et al., 2007; Goldberg et al., 2008) or depolarizing their activation voltage to make them unactivated prior to spike initiation (Wester and Contreras, 2013) could both result in a loss of the inverse correlation between spike threshold and *dV*/*dt*.

In addition to modulating threshold dynamic, the biophysical properties of membrane currents could also control neuronal spike initiation (Koch, 1999; Izhikevich, 2005; Prescott and Sejnowski, 2008; Prescott et al., 2008a,b; Yi et al., 2014a,b). It is shown that if the K^+^ current that flows out of the cell is absent or unactivated at the potentials around spike threshold, i.e., perithresholds, the neuron generates a continuous frequency-current curve through a saddle-node on invariant circle (SNIC) bifurcation, i.e., Hodgkin class 1 excitability (Izhikevich, 2005; Prescott et al., 2008a,b; Yi et al., 2014a). On the contrary, if the outward K^+^ current has already activated at the perithresholds, the neuron generates a discontinuous frequency-current curve through a Hopf bifurcation, i.e., Hodgkin class 2 excitability (Izhikevich, 2005; Prescott et al., 2008a,b; Yi et al., 2014a). Furthermore, Rothman and Manis (2003a,b,c) find that a high density of low-threshold K^+^ current in ventral cochlear nucleus is responsible for phasic firing of class 2 excitability, while a lower density promotes regular firing of class 1 excitability. These reports suggest that membrane biophysics is able to further determine neuronal input-output relations. Then, the dynamics of the spike threshold should also be dependent on input-output properties. Uncovering the biophysical connection between them is crucial for explaining how biophysical properties contribute to neural coding. Meanwhile, it could also provide a deeper insight into the mechanism of neural coding than a purely phenomepological description of input-output relation. However, the relevant studies are still lacking.

In fact, the biophysical properties of membrane currents not only affect spike threshold dynamic and input-output relation, but also influence neuronal energetics. During the generation of action potential, there is flux of different ions across their voltage-gated ionic channels, such as, influx of Na^+^ and efflux of K^+^. In this process, the ions need to expand significant quantities of energy to permeate cell membrane against their concentration gradient (Attwell and Laughlin, 2001; Niven and Laughlin, 2008; Alle et al., 2009; Carter and Bean, 2009; Sengupta et al., 2010, 2013, 2014; Moujahid et al., 2011, 2014; Moujahid and D'Anjou, 2012). The influx or efflux of ions, i.e., inward or outward ionic currents, dominate and make a significant contribution to neuronal energy consumption (Attwell and Laughlin, 2001; Alle et al., 2009; Sengupta et al., 2010, 2013, 2014). Previous studies (Alle et al., 2009; Carter and Bean, 2009; Sengupta et al., 2010, 2013; Moujahid and D'Anjou, 2012; Moujahid et al., 2014) have shown that adjusting the biophysical properties of voltage-gated Na^+^ and K^+^ currents, such as, channel conductance or activation/inactivation time constant, could modulate the energy efficiency of neuron. Then, a critical question arises as to how the spike threshold dynamic, a basic property of neuron, influences its energy consumption. Until now, there is still no relevant research on this issue.

Here, we systematically characterize the input-output property and energy efficiency of the neuron with different spike threshold dynamics. To achieve this goal, we first adopt a two-dimensional biophysical model and vary its parameter that controls the voltage-dependency of K^+^ current to produce different relationships between spike threshold and *dV*/*dt*. Then, we investigate how the minimal neuron responds to external stimulus as well as its relevant biophysical mechanism in the case of different threshold dynamics. Finally, we deduce the energy functions involved in the dynamics of neuron model, and determine the energy efficiency associated with each threshold dynamic.

## Materials and methods

### Two-dimensional neuron model

A two-dimensional biophysical model proposed by Prescott et al. (2008a) is adopted to explore how spike threshold dynamic modulates neuronal input-output relation and metabolic energy in present study. It is a modified version of Morris-Lecar model, which incorporates three ionic currents, i.e., a fast Na^+^ current *I*_*Na*_, a delayed rectifying K^+^ current *I*_*K*_, as well as a leak current *I*_*L*_. The model is given by the following differential equations (Prescott et al., 2008a)

(1) $$\begin{array}{l} C\frac{dV}{dt}=I_{in}+I_{noise}-{\bar{g}}_{K}n(V-V_{K})-{\bar{g}}_{Na}m_{\infty }\\ \;(V)(V-V_{Na})-g_{L}(V-V_{L}) \end{array}$$

(2) $$\frac{dn}{dt}=\varphi_{n}\frac{n_{\infty }(V)-n}{\tau_{n}(V)}$$

where *V* is the membrane voltage and *n* is the activation gating variable for *I*_*K*_. The three terms on the right side of Equation (1), i.e., *g*_*K*_ *n*(*V* − *V*_*K*_), *g*_*Na*_ *m*_∞_ (*V*)(*V* − *V*_*Na*_) and *g*_*L*_ (*V* − *V*_*L*_), respectively denote slow outward *I*_*K*_, fast inward *I*_*Na*_ and outward $I_{L}\;.\;m_{\infty }(V)=0.5\left\{1+\tanh\left[\left(V-\beta_{m}\right)/\gamma_{m}\right]\right\}$ and $n_{\infty }(V)=0.5\left\{1+\tanh\left[\left(V-\beta_{n}\right)/\gamma_{n}\right]\right\}$ are the steady-state voltage-dependent activation functions for *I*_*Na*_ and *I*_*K*_, and τ_*n*_ (*V*) = 1/cosh [(*V* − β_*n*_)/2γ_*n*_] is the K^+^ voltage-dependent time constant function. The kinetics of inward *I*_*Na*_ are controlled by parameter β_*m*_ and γ_*m*_, and the kinetics of outward *I*_*K*_ are controlled by β_*n*_ and γ_*n*_. In previous modeling study, Wester and Contreras (2013) have shown that hyperpolarizing K^+^ activation voltage, even in the absence of Na^+^ inactivation, is sufficient to produce a dynamic spike threshold that is inverse to the preceding *dV*/*dt*. Then, we vary parameter β_*n*_ from −5 to −15 mV in steps of −2 mV to produce different sensitivity of spike threshold to *dV*/*dt* in our stimulation. These values of β_*n*_ can span different spike initiation dynamics of the model (Prescott et al., 2008a). Table 1 gives the numerical values and corresponding neural functions of the parameters in two-dimensional model, which are the same as those described in Prescott et al. (2008a).

**Table 1:** **Parameters in two-dimensional model (Prescott et al., 2008a)**.

**Symbol**	**Value**	**Description**
*C*	2μF/cm^2^	Membrane capacitance
*g*_*Na*_	20mS/cm^2^	Na^+^ maximal conductance
*g*_*K*_	20mS/cm^2^	K^+^ maximal conductance
*g*_*L*_	2mS/cm^2^	Leak maximal conductance
*V*_*Na*_	50 mV	Na^+^ reversal potential
*V*_*K*_	−100 mV	K^+^ reversal potential
*V*_*L*_	−70 mV	Leak reversal potential
β_*m*_	−1.2 mV	Controlling the half-activation voltage of Na^+^ current
γ_*m*_	18 mV	Slope factor of activation curve *m*_∞_ (*V*)
β_*n*_	−5, −7, −9, −11, −13, or −15 mV	Controlling the half-activation voltage of K^+^ current
γ_*n*_	10 mV	Slope factor of activation curve *n*_∞_ (*V*)
φ_*n*_	0.15 (unitless)	Scaling factor for K^+^ activation variable *n*

*I*_*in*_ is the injected current used to stimulate neuron, which can be either steps or ramps in our study. *I*_*noise*_ is used to replicate synaptic noise, and is modeled as an Ornstein-Uhlenbeck process (Uhlenbeck and Ornstein, 1930)

(3) $$\frac{dI_{noise}}{dt}=-\frac{I_{noise}}{\tau_{noise}}+\sigma N(t)$$

where *N*(*t*) is a random number drawn from a Gaussian distribution with average 0 and unit variance. The amplitude of weak noise *I*_*noise*_ is controlled by the scaling parameter σ (Destexhe et al., 2001; Prescott and Sejnowski, 2008; Prescott et al., 2008a,b), which could vary from 0μA/cm^2^ to 3μ A/cm^2^ in our study. The time constant is τ_*noise*_ = 5ms (Prescott and Sejnowski, 2008; Prescott et al., 2008b). When we determine spike threshold, phase response curve (PRC) and bifurcation patterns, the noisy current is removed from the neuron.

### Method to calculate spike threshold

The spike threshold for different values of *dV*/*dt* is determined by a novel approach proposed by Wester and Contreras (2013). According to their description, we use *I*_*in*_ to produce a cluster of ramps to stimulate the neuron, so

(4) $$I_{in}=\{\begin{array}{l} Kt\;(0\leq t\leq t_{0})\\ 0\;(t>t_{0}) \end{array}$$

The ramp slope *K* controls the values of *dV*/*dt* leading to the spike initiation. With a larger value of *K*, the membrane potential *V* is forced to approach the threshold potential at a faster speed, which corresponds to a bigger value of *dV*/*dt*. The stimulation duration is controlled by *t*_0_. For a given slope *K*, the membrane potential *V* will gradually approach the threshold as *t*_0_ increases. When membrane potential *V* is around the threshold potential, we stepwise extend ramp duration *t*_0_ to make each step result in about additional 0.1 mV depolarization in *V* until an action potential is initiated in the neuron. In this way, if *V* is driven to cross spike threshold at the time of ramp offset, there will be a spike generated after removing ramp (i.e., *t* > *t*_0_). Conversely, the neuron fails to initiate a spike if *V* does not reach the threshold potential at the time of ramp offset. Then, we empirically increase ramp duration *t*_0_ to seek such a special membrane potential V^*^ : 0.1 mV hyperpolarized to V^*^ is subthreshold and neuron fails to initiate a spike at the time of ramp offset, whereas 0.1 mV depolarized to V^*^ is suprathreshold and neuron could initiate a spike at the time of ramp offset. We define this special membrane potential V^*^ as the spike threshold of the neuron. In this manner, the upstroke of the spike is purely due to the sufficient activation of Na^+^ current, which has nothing to do with the current ramp. This method allows us to measure the spike threshold with a high precision less than 0.1 mV.

### Phase response curve calculation

The PRC measures the phase shift of a periodically oscillating neuron in response to a brief current pulse delivered at different phases of the oscillation cycle (Ermentrout, 1996; Izhikevich, 2005; Smeal et al., 2010; Fink et al., 2011; Schultheiss et al., 2012). The PRC of the neuron can be defined as (Ermentrout, 1996; Izhikevich, 2005; Smeal et al., 2010; Schultheiss et al., 2012)

(5) $$PRC(\vartheta )=1-T^{\prime }(\vartheta )/T$$

where *T* is the oscillation period of the neuron without perturbation (i.e., 1/*T* represents natural oscillation frequency), and *T*′(ϑ) is the oscillation period when the neuron is stimulated at phase ϑ. A positive value of PRC indicates there is a phase advance, and a negative value indicates a phase delay. If the amplitude of current pulse is sufficiently small and its duration is sufficiently brief, the PRC becomes the infinitesimal PRC, which could reflect the intrinsic dynamics of the oscillator (Ermentrout, 1996; Smeal et al., 2010; Fink et al., 2011; Schultheiss et al., 2012). In the following, we use “PRC” to refer to the infinitesimal PRC. Further, the PRCs of neural oscillator have often been classified into two categories: Type I that respond with only phase advances to excitatory stimuli, and Type II that display both phase advances and delays (Hansel et al., 1995; Smeal et al., 2010; Fink et al., 2011).

### Method to determine energy consumption in two-dimensional model

We use the method proposed by Moujahid et al. (2011, 2014) and Moujahid and D'Anjou (2012) to determine the electrochemical energy involved in the modified Morris-Lecar model. The model in Equation (1) can be regarded as an electrical circuit, which consists of membrane capacitance *C*, Na^+^, K^+^ and leak ionic channels. According to the description by Moujahid et al. (2011, 2014) and Moujahid and D'Anjou (2012), the total electrical energy accumulated in this circuit at a given time can be expressed by

(6) $$E(t)=\frac{1}{2}CV^{2}+E_{Na}+E_{K}+E_{L}$$

Here, $\frac{1}{2}$*CV*^2^ is the electrical energy accumulated in the membrane capacitance. *E*_*Na*_, *E*_*K*_, and *E*_*L*_ are the energies in the batteries needed to create the concentration jumps in Na^+^, K^+^ and chloride, respectively. These energies could be supplied by external stimuli, i.e., *I*_*in*_ or *I*_*noise*_. The first-order derivative with respect to time of the Equation (6) is

(7) $$\frac{dE}{dt}=CV\frac{dV}{dt}+I_{Na}V_{Na}+I_{K}V_{K}+I_{L}V_{L}$$

Substituting $\frac{dV}{dt}$ with Equation (1), the energy rate δ (i.e., $\frac{dE}{dt}$) in the circuit can be written as

(8) $$\delta =(I_{in}+I_{noise})V-I_{Na}(V-V_{Na})-I_{K}(V-V_{K})-I_{L}(V-V_{L})$$

where (*I*_*in*_ + *I*_*noise*_)*V* is the energy power supplied by stimulus. The last three terms on the right hand of Equation (8) represent the energy consumption rate of the ionic channels. If we substitute *I*_*Na*_, *I*_*K*_, and *I*_*L*_ with their expressions, we can deduce the energy rate of each ionic channel

(9) $$\delta_{Na}={\bar{g}}_{Na}m_{\infty }(V){\left(V-V_{Na}\right)}^{2}$$

(10) $$\delta_{K}={\bar{g}}_{K}n{\left(V-V_{K}\right)}^{2}$$

(11) $$\delta_{L}=g_{L}{\left(V-V_{L}\right)}^{2}$$

It is easy to see that this method is not based on the stoichiometry of the ions. Thus, it requires no hypothesis about the overlapping between Na^+^ and K^+^ ions, and then avoids the overestimate values of energy (Moujahid et al., 2011, 2014; Moujahid and D'Anjou, 2012).

### Numerical stimulation

The differential equations of the entire system are numerically integrated with MATLAB. The bifurcation analysis is performed with XPPAUT (Ermentrout, 2002) following the standard procedures. In bifurcation analysis, we use *I*_*in*_ to produce step currents to stimulate the neuron and systematically vary its intensity to determine at what point the neuron qualitatively changes its dynamical behavior, such as, starting or ceasing repetitive spiking. This special point corresponds to a bifurcation. Further, the PRC is also calculated by XPPAUT.

## Results

**Figure 1:**
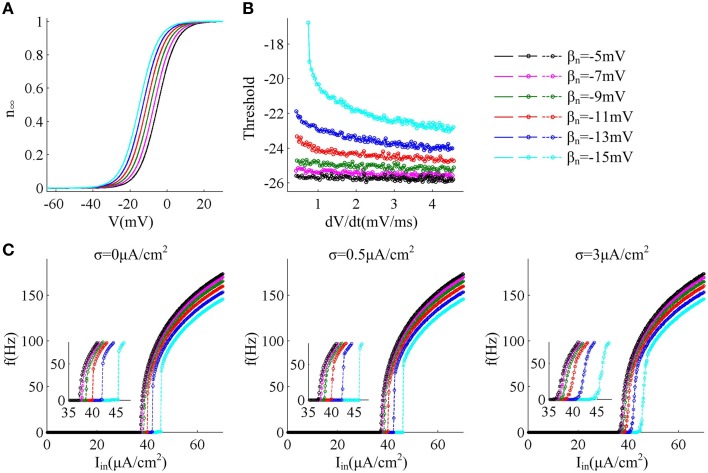
***f*** **−** ***I***_***in***_ **curves associated with different threshold dynamics induced by adjusting β**_***n***_. **(A)** The half-activation voltage β_*n*_ of activation variable *n* is hyperpolarized from −5 to −15mV with a step of −2mV. **(B)** Spike threshold as a function of *dV*/*dt* with different values of β_*n*_. The range of *dV*/*dt* is from 0.45 to 4.5mV/ms. **(C)** *f* − *I*_*in*_ curves generated by the neuron with different threshold dynamics for three levels of noise. The noise amplitude is σ = 0, 0.5, and 3μA/cm^2^, respectively.

In this section, we first adjust parameter β_*n*_ that controls the half-activation voltage of K^+^ channel to produce the spike threshold that has different sensitivity to the preceding *dV*/*dt*, as shown in Figures 1A,B. One can find that the spike threshold becomes more depolarized as we shift β_*n*_ alone from −5 to −15mV in steps of −2mV (Figure 1B). For three cases of β_*n*_ = −5, −7, and −9mV, the spike thresholds are all insensitive to *dV*/*dt*, and there is always no inverse relationship between spike threshold and *dV*/*dt*. On the contrary, the spike threshold shows relatively large variations and becomes sensitive to *dV*/*dt* with β_*n*_ = −11, −13, and −15mV. In these three cases, the spike threshold varies inversely with the preceding *dV*/*dt*, and simultaneously the inverse relationship becomes more significant as β_*n*_ decreases. The range of *dV*/*dt* in Figure 1B is from 0.45 to 4.5mV/ms, which is achieved by increasing ramp slope *K* in Equation (4). This range is selected in accordance with previous modeling (Wester and Contreras, 2013) and experimental (Wilent and Contreras, 2005) studies. In the following, we respectively explore neuronal input-output relation and energy efficiency in these six cases.

### Input-output property of the neuron with different threshold dynamics

For different sensitivity of spike threshold to *dV*/*dt*, we respectively investigate how neuron responds to constant current in the cases of no noise (σ = 0μA/cm^2^), low noise (σ = 0.5μ A/cm^2^) and high noise (σ = 3μ A/cm^2^). To achieve this goal, we use *I*_*in*_ to produce step current to stimulate the neuron and systematically alter its intensity to determine neuronal spike frequency *f*.

Figure 1C gives neuronal spike frequency *f* as a function of input current *I*_*in*_ (i.e., *f* − *I*_*in*_ curve) in six cases of threshold dynamic. For three levels of noise, one can observe that the depolarization of spike threshold slightly reduces the slope of *f* − *I*_*in*_ curve at the low firing rates and obviously shifts the curve to the right, which corresponds to increasing the minimal current intensity used for triggering repetitive spike (i.e., current threshold). If spike threshold is insensitive to *dV*/*dt* (i.e., β_*n*_ = −5, −7, and −9mV), the neuron could spike repetitively at very low frequencies in all levels of noise, which endows it with a continuous *f* − *I*_*in*_ curve. However, when spike threshold is sensitive to *dV*/*dt* (i.e., β_*n*_ = −11, −13, and −15mV), the neuron is unable to maintain repetitive spike at low rates and produces a discontinuous *f* − *I*_*in*_ curve in the cases of no or low noise levels (Figure 1C). This discontinuous *f* − *I*_*in*_ curve could be switched to continuous by high level of noise.

**Figure 2:**
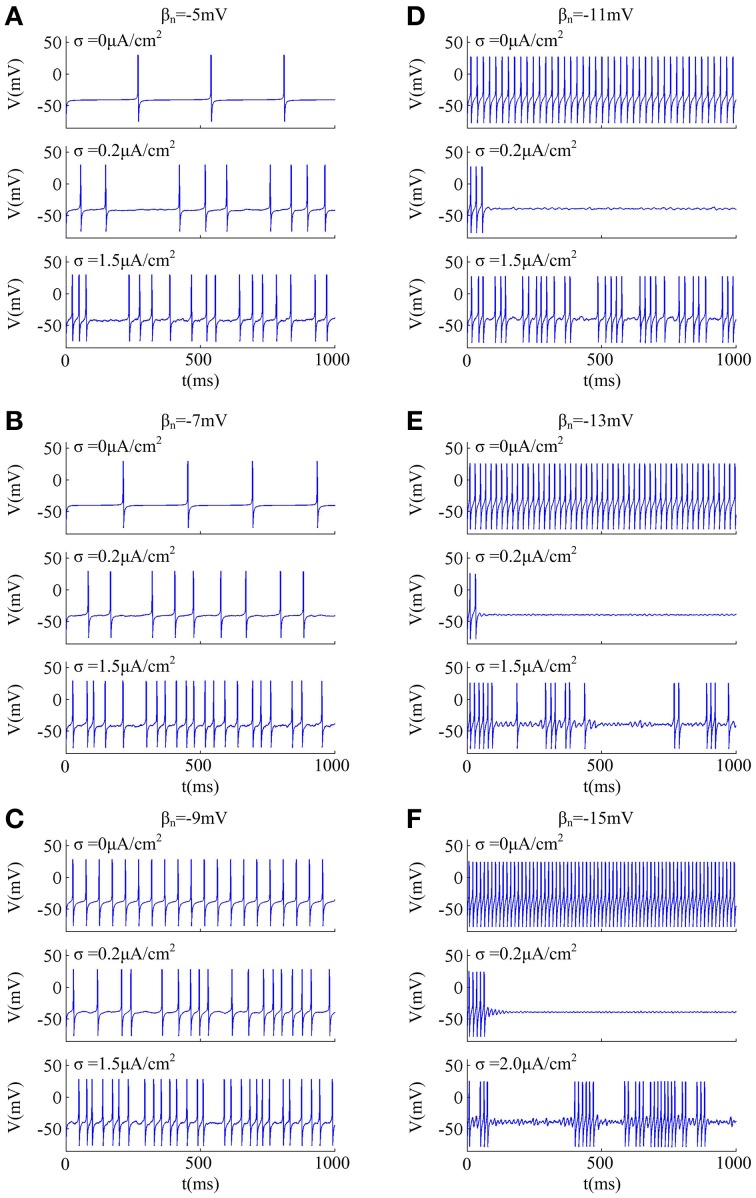
**Effects of weak noise on spiking trains around the bifurcation**. The input current is **(A)** *I*_*in*_ = 37.3μ A/cm^2^, **(B)** *I*_*in*_ = 37.8μ A/cm^2^, **(C)** *I*_*in*_ = 38.72μ A/cm^2^, **(D)** *I*_*in*_ = 40.2μ A/cm^2^, **(E)** *I*_*in*_ = 42.2μ A/cm^2^, and **(F)** *I*_*in*_ = 45.8μ A/cm^2^. The values of noise amplitude σ have been indicated in each panel.

**Figure 3:**
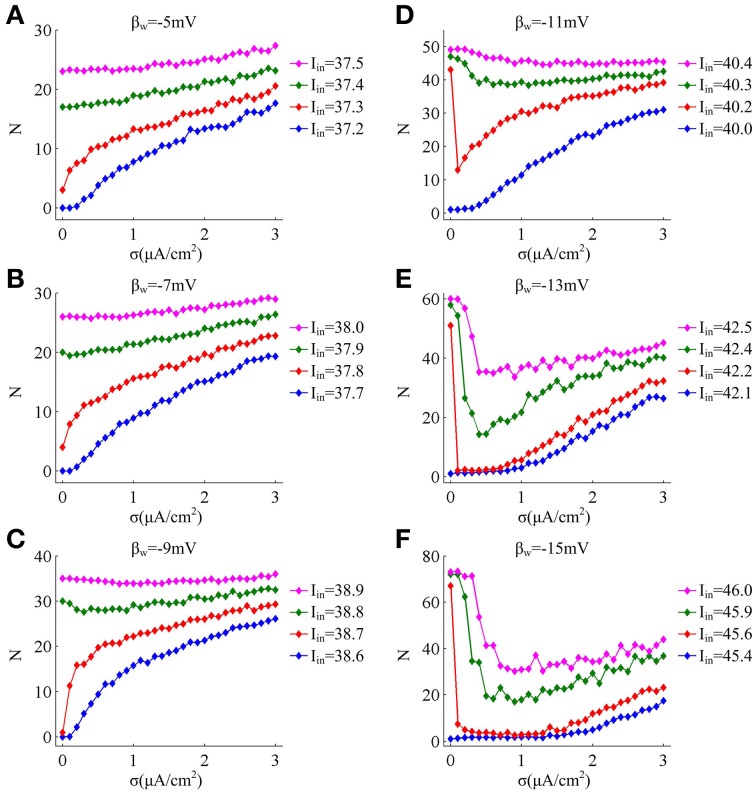
**Mean numbers of spikes as a function of noise amplitude for each threshold dynamic. (A–F)** respectively give the mean spike number *N* (40 trials) as noise amplitude σ is increased in the neuron for 1000 ms time interval with different values of β_*n*_. The value of *I*_*in*_ indicated by blue line is below the bifurcation point I^*^_in_ and there is no repetitive spiking generated in the neuron without noise, while the values of *I*_*in*_ indicated by three other colors are above the bifurcation point I^*^_in_.

Since noise is another ubiquitous feature of the nervous system with myriad effects on neural coding (Tuckwell, 1989; Gerstner and Kistler, 2002; Tuckwell et al., 2009; Tuckwell and Jost, 2010), we further investigate how noise modulates spike trains of the neuron with different spike threshold dynamics, as shown in Figures 2, 3. It is observed that no matter there is an inverse relationship between spike threshold and *dV*/*dt* or not, the spike number always increases monotonically from 0 as noise amplitude σ increases when *I*_*in*_ is less than the bifurcation value I^*^_in_. For *I*_*in*_ just beyond I^*^_in_, the noise could inhibit or even terminate the repetitive spiking of neuron when its spike threshold is sensitive to *dV*/*dt* (Figures 2D–F). In this case, the neuron is able to generate repetitive spike without noise (i.e., σ = 0μ A/cm^2^), since *I*_*in*_ has already exceeded bifurcation value I^*^_in_. Introducing synaptic noise makes the spike trains become irregular. Unexpectedly, weak noise (such as, σ = 0.2μ A/cm^2^) has an obvious inhibitory effect on neuronal spiking behavior, which even terminates repetitive spiking for a long time. When noise amplitude is increased to σ = 1.5μ A/cm^2^ or even higher, there will be more spikes evoked again. That is, when *I*_*in*_ is in the vicinity of I^*^_in_, small noise could noticeably inhibit neuronal spiking and there is a minimum in the mean spike number as σ goes up (Figures 3D–F). Meanwhile, as the inverse relationship between spike threshold and *dV*/*dt* gets pronounced, the inhibitory effect induced by small noise becomes stronger. However, this inhibitory effect does not appear in the neuron with an insensitive spike threshold to *dV*/*dt* (left panels, Figures 2, 3). In this case, the noise only disturbs its spike trains and makes them become irregular, which is unable to terminate repetitive spiking (Figures 2A–C).

### Phase response curves of the neuron with different threshold dynamics

In previous section, we have found that different sensitivity of spike threshold to *dV*/*dt* could result in distinct (i.e., discontinuous or continuous) *f* − *I*_*in*_ curves in the case of no or low noise. In this section, we use PRC theory to further characterize neuronal response properties in the case of different threshold dynamics.

**Figure 4:**
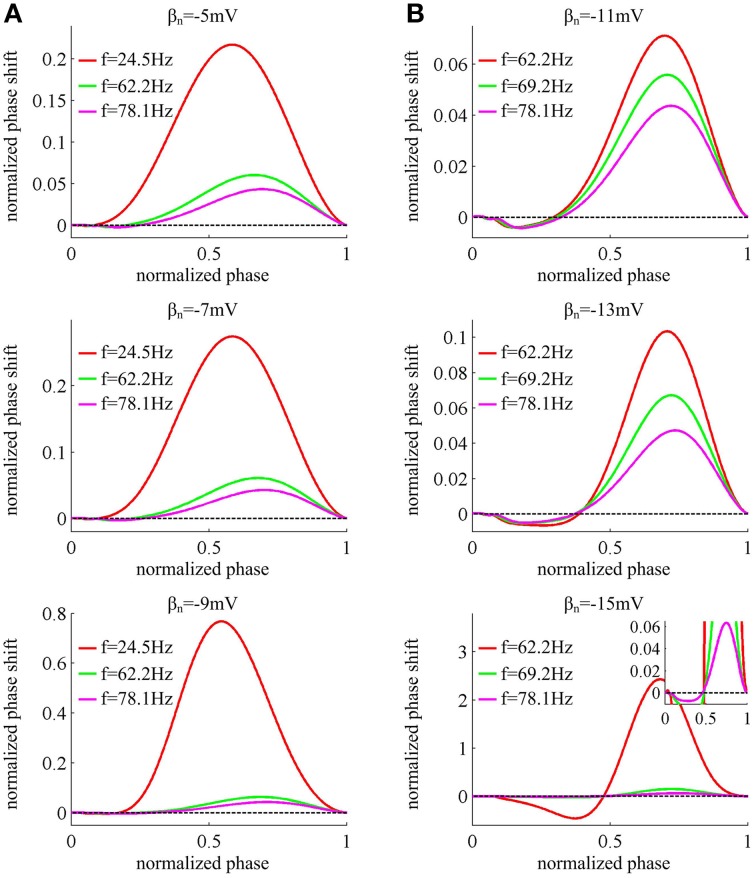
**PRCs of the neuron with different threshold dynamics**. The neurons in **(A)** have an insensitive spike threshold to *dV*/*dt*, and in **(B)** have a sensitive threshold to *dV*/*dt*. For each threshold dynamic, we compute neuronal PRC at three different natural firing frequencies. The corresponding stimulation current is: *I*_*in*_ = 37.52, 39.31, and 40.87μ A/cm^2^ for β_*n*_ = −5mV; *I*_*in*_ = 37.966, 39.70, and 41.33μ A/cm^2^ for β_*n*_ = −7mV; *I*_*in*_ = 38.74, 40.28, and 41.94μ A/cm^2^ for β_*n*_ = −9mV; *I*_*in*_ = 41.17, 41.81, and 42.87μ A/cm^2^ for β_*n*_ = −11mV; *I*_*in*_ = 42.63, 43.20, and 44.25μ A/cm^2^ for β_*n*_ = −13mV; *I*_*in*_ = 45.478, 45.688, and 46.5μ A/cm^2^ for β_*n*_ = −15mV. All PRCs are computed in the case of no noise, i.e., σ = 0μ A/cm^2^.

Figure 4 displays the PRCs of the neuron model in six cases of spike threshold dynamic. It is found that the PRC is dependent on the natural oscillation frequency of neuron, and increasing it could attenuate the amplitude of phase shift. When spike threshold is insensitive to *dV*/*dt*, the neuron generates type I PRC, which exclusively displays phase advances (i.e., positive values) to excitatory brief pulse (Figure 4A). However, when spike threshold has an obvious inverse relation with *dV*/*dt*, the neuron shows phase delays (i.e., negative values) at earlier phases and phase advances at later phases (Figure 4B), which is manifested as a type II PRC. It has been proposed that type I PRC corresponds to a continuous *f* − *I*_*in*_ curve and type II PRC corresponds to a discontinuous *f* − *I*_*in*_ curve (Ermentrout, 1996; Izhikevich, 2005; Smeal et al., 2010; Fink et al., 2011). Our simulation results in Figures 1C, 4 are in accordance with this proposal. Further, it is worth pointing out that there are very small negative regions at the earlier phases of type I PRCs (Figure 4A). This is because the action potentials generated in Morris-Lecar like model consume a much larger portion of interspike interval than other models (Rinzel and Ermentrout, 1998; Fink et al., 2011). But according to the descriptions of Fink et al. (2011), we could ignore these early small phase delays in type I PRCs.

### Biophysical basis of the spike initiation associated with different threshold dynamics

By varying parameter β_*n*_, we have identified the input-output property associated with each spike threshold dynamic. Our next step is to explore why the neuron with distinct threshold dynamics produces different input-output properties. It has been known that the membrane currents with opposite directions play different roles in spike generation. The currents flowing into the cell mainly depolarize membrane voltage to produce the rapid upstroke of the spike (i.e., positive feedback), whereas the currents flowing out of the cell mainly hyperpolarize membrane voltage which are responsible for the repolarization and produce the downstroke of the spike (i.e., negative feedback) (Izhikevich, 2005; Prescott et al., 2008a,b; Yi et al., 2014a). Here, we investigate how the opposite currents interact at the perithreshold potentials to determine neuronal response property in six cases of spike threshold dynamic.

**Figure 5:**
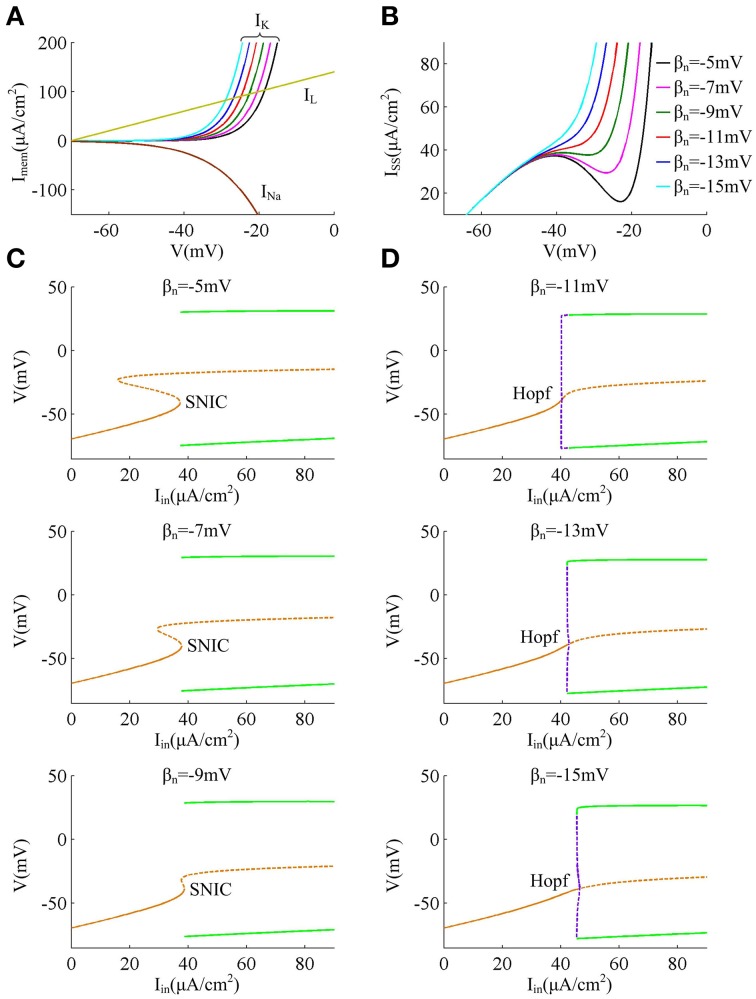
**Biophysical basis of the spike initiation for different threshold dynamics. (A)** shows the individual steady-state membrane currents at the subthreshold potentials. Decreasing β_*n*_ has no effects on the activations of inward *I*_*Na*_ and outward *I*_*L*_, while it causes outward *I*_*K*_ to be more strongly activated by perithreshold depolarization. **(B)** gives the relationship between steady-state net membrane current *I*_*SS*_ and membrane potential *V* (i.e., *I*_*SS*_ − *V* curve). *I*_*SS*_ is computed as the sum of three individual currents, i.e., *I*_*SS*_ = *I*_*Na*_ + *I*_*K*_ + *I*_*L*_. **(C,D)** summarize the bifurcation diagram associated with each spike threshold dynamic. The stable equilibrium is indicated by orange solid line and unstable is orange dotted line. The stable limit cycle is indicated by green solid line and unstable is purple dotted line.

Reducing parameter β_*n*_ from −5 to −15mV results in a hyperpolarizing shift in the half-activation voltage of outward K^+^ current *I*_*K*_ (Figure 1A), which causes *I*_*K*_ to be more strongly activated by the perithreshold depolarization (Figure 5A). For three cases that the spike threshold is insensitive to *dV*/*dt* (i.e., β_*n*_ = −5, −7, and −9mV), the outward *I*_*K*_ activates at a higher potential than inward *I*_*Na*_ (Figure 5A), which indicates that the slow outward current *I*_*K*_ does not become activated until after the spike is initiated. In these three cases, the relationship between steady-state net membrane current *I*_*SS*_ and membrane voltage *V* (i.e., *I*_*SS*_ − *V* curve) is always non-monotonic (Figure 5B), which has a region of negative slope. At the local maximum of *I*_*SS*_ − *V* curve, the inward *I*_*Na*_ balances outward unactivated *I*_*K*_ and outward *I*_*L*_. Then, any further depolarization could result in the progress activation of *I*_*Na*_ and make it become self-sustaining to generate the upstroke of the spike. In other words, the bifurcation occurs at this voltage, i.e., ∂ *I*_*SS*_ /∂ *V* = 0. Since the depolarizing current *I*_*Na*_ faces no restraint of hyperpolarizing current at the perithreshold potentials, the membrane potential *V* could be driven to slowly pass through spike threshold. Thus, the neuron is able to spike repetitively at low frequencies and produce a continuous *f* − *I*_*in*_ curve. This continuous input-output property is generated through a SNIC bifurcation (Figure 5C), which corresponds to a non-monotonic *I*_*SS*_ − *V* curve (Izhikevich, 2005; Prescott et al., 2008a,b; Yi et al., 2014a). Further, because inward *I*_*Na*_ dominates spike initiation without the restraint of *I*_*K*_ at the perithresholds, a brief, excitatory stimulus only leads to advances in oscillation cycle and positive values of phase shift, which corresponds to a type I PRC.

For the other three cases that the spike threshold is sensitive to *dV*/*dt* (i.e., β_*n*_ = −11, −13, and −15mV), the outward *I*_*K*_ activates at roughly the same *V* with inward *I*_*Na*_ or at a slightly lower *V* than *I*_*Na*_ (Figure 5A). The activation of *I*_*K*_ at low potentials makes the outward currents become so strong that the inward *I*_*Na*_ is unable to balance them at the perithreshold potentials, which results in a monotonic *I*_*SS*_ − *V* curve without local maximum (Figure 5B). To initiate action potentials, the inward *I*_*Na*_ must exploit its fast kinetic to activate faster than slow outward *I*_*K*_, and drives *V* through threshold potential with a sufficient speed that the outward *I*_*K*_ cannot catch up. Only in this way can the positive feedback outrun negative feedback to produce the upstroke of the spike. Since the *V* trajectory between two spikes must be more rapid than *I*_*K*_, the neuron is unable to spike repetitively at low frequencies, which endows it with a discontinuous *f* − *I*_*in*_ curve. This discontinuous input-output property is generated through a Hopf bifurcation (Figure 5D), which corresponds to a monotonic *I*_*SS*_ − *V* curve (Izhikevich, 2005; Prescott et al., 2008a,b; Yi et al., 2014a). Further, in this case there is a special subthreshold region where the activation of low-threshold *I*_*K*_ is greater than inward *I*_*Na*_. When voltage trajectory pass through this region, an excitatory pulse will evoke a larger response from outward *I*_*K*_ than from inward *I*_*Na*_, which leads to negative PRC values at early phases. At higher membrane potential later in this special subthreshold region, the fast activating *I*_*Na*_ dominates neuronal response to brief excitatory pulse, which leads to the positive PRC values at later phases. Then, the neuron generates a type II PRC that has both phase delays and advances in these three cases.

Further, as spike threshold gets depolarized, the outward *I*_*K*_ becomes more strongly activated at the perithreshold potentials, which increases the net current *I*_*SS*_ and makes it reach a higher outward level prior to spike initiation. Since the outward current hyperpolarizes membrane potential *V* and prohibits action potential, there should be stronger step current *I*_*in*_ to counteract outward current and activate inward *I*_*Na*_ to generate spike. Then, the current threshold for triggering neuronal repetitive spiking increases as spike threshold gets depolarized.

Finally, when Hopf bifurcation occurs (i.e., the spike threshold is sensitive to *dV*/*dt*), there is a narrow bistable region in the vicinity of bifurcation, where stable resting state and stable limit cycle coexist (Figure 5D). Then, synaptic noise could switch voltage trajectory from one attractor, a stable limit cycle, to another, a stable resting point (Tuckwell et al., 2009; Tuckwell and Jost, 2010, 2011, 2012; Guo, 2011). This is the basis of the inhibitory effects of weak noise on spiking behavior. Meanwhile, the bistable region widens as the relationship between spike threshold and *dV*/*dt* gets pronounced, which causes the inhibitory effects of weak noise on repetitive spiking to become stronger. On the contrary, there is no bistable region in the case of SNIC bifurcation (Figure 5C), so the noise is unable to inhibit or terminate neuronal spiking in this case, i.e., the spike threshold is insensitive to *dV*/*dt*.

### Energy efficiency in the neuron with different threshold dynamics

We have identified the input-output property and spike initiation mechanism associated with each threshold dynamic. Here, we characterize the energy efficiency consumed by the neuron in six cases of threshold dynamic.

**Figure 6:**
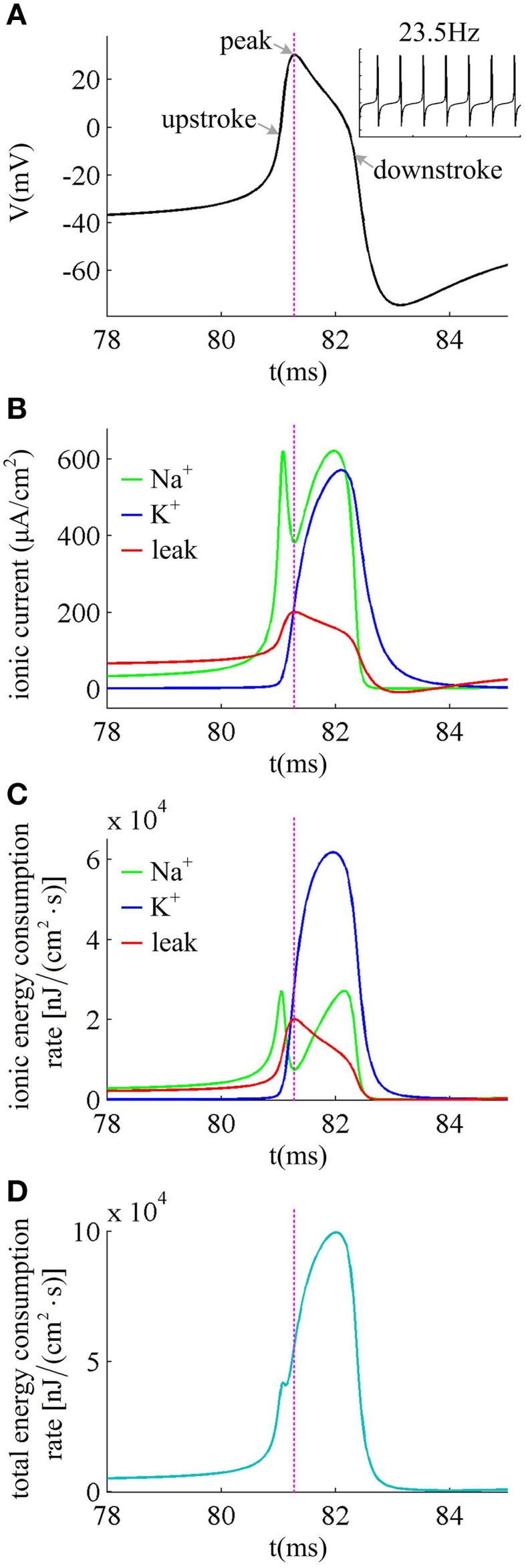
**Ionic currents and energy consumption involved in a spike. (A)** shows an action potential generated in the neuron with β_*n*_ = −5mV. **(B)** gives the Na^+^, K^+^ and leak currents in this action potential. The Na^+^ current is negative but we plot it with a positive sign. **(C)** shows the energy consumption rate for each ionic current, and **(D)** gives the total energy consumption rate of the action potential. The stimulus is *I*_*in*_ = 37.5μ A/cm^2^ and σ = 0μ A/cm^2^. In this case, the neuron generates repetitive spiking at about 23.5 Hz.

We first describe how ionic currents and their energy consumption evolve during the generation of a spike. Figure 6A shows an action potential generated in the neuron with β_*n*_ = −5mV to *I*_*in*_ = 37.5μ A/cm^2^ in the case of no noise (i.e., σ = 0μ A/cm^2^). At this value of *I*_*in*_ and σ, the neuron spikes repetitively at about 23.5 Hz. Figure 6B gives the Na^+^, K^+^ and leak currents corresponding to the spike waveform described in Figure 6A. The Na^+^ current flows into the cell and has a negative sign, but we plot it with a positive sign for a better visualization of the overlap between Na^+^ and K^+^ currents. During the upstroke, the Na^+^ current first activates and drives membrane voltage to quickly depolarize. Then, the outward K^+^ current activates which hyperpolarizes membrane voltage and leads to the downstroke. The energy consumption rates of the three ions are shown in Figure 6C, which are computed according to Equations (9)–(11). They represent the instantaneous energy consumption per second by corresponding ionic channel, which are all positive. One can observe that there are overlaps between Na^+^ and K^+^ energy, especially during the downstroke (Figure 6C). Figure 6D gives the total energy rate δ consumed by all the ionic currents, which is used to generate the action potential in Figure 6A. In order to maintain the spiking activity of the neuron, this energy consumption must be replenished by the ion pumps and metabolically supplied by the hydrolysis of ATP molecules.

**Figure 7:**
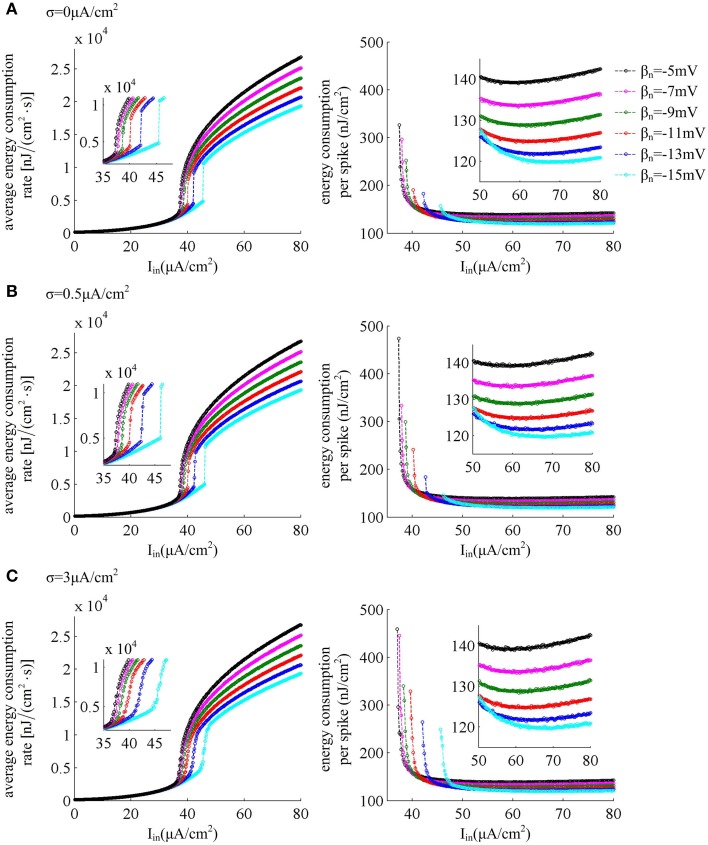
**Energy consumption as a function of** ***I***_***in***_ **associated with each threshold dynamic**. Left panels give the average energy consumption rate of the neuron with different spike threshold dynamics for three levels of noise. The energy consumption rate is averaged over the 7000 ms time interval. Right panels are the total electrochemical energy consumed by an action potential related to each spike threshold dynamic and input current *I*_*in*_. The noise amplitude is **(A)** σ = 0μ A/cm^2^, **(B)** σ = 0.5μ A/cm^2^, and **(C)** σ = 3μ A/cm^2^.

The left panels in Figure 7 give the average energy consumption rate δ as a function of input current *I*_*in*_ (i.e., δ − *I*_*in*_ curve) in six cases of spike threshold dynamics for three levels of noise. It can be found that the energy consumption rate δ in quiescent state is much lower than that in spiking state. This is because the increase of supplied energy to the neuron, i.e., increasing step current, promotes the ionic to pass through cell membranes, and makes them consume more energy. When spike threshold is insensitive to *dV*/*dt* (i.e., β_*n*_ = −5, −7, and −9mV), the δ − *I*_*in*_ curve is always continuous for three levels of noise. However, if there is an obvious inverse relation between threshold and *dV*/*dt* (i.e., β_*n*_ = −11, −13, and −15mV), the δ − *I*_*in*_ curve is discontinuous in the cases of no or low noise and continuous for high level of noise. Thus, the energy consumption rate of the neuron during the transition from quiescent state to spiking regime is dependent on its firing rates, which is displayed in Figure 1C. As spike threshold gets depolarized, the δ − *I*_*in*_ curve in firing regime shifts to the right and the corresponding average energy consumption rate δ of the neuron decreases.

The right panels in Figure 7 show the total energy consumption in nJ per cm^2^ calculated as the integral over long period of time of the area under the instantaneous ionic channel energy curve [i.e., the sum of the energy rates given by Equations (9)–(11)] divided by the number of spikes, which gives the energy consumption of a single spike. As step current *I*_*in*_ increases, the energy consumed in one spike first quickly decreases, and then has a very slight increase (about 0.1nJ/cm^2^ per 1μ A/cm^2^). As threshold gets depolarized, the energy consumption in one action potential becomes larger with some low *I*_*in*_ values, and the synaptic noise obviously increases this consumption. However, with high values of *I*_*in*_, the energy demand for a spike gets smaller as spike threshold depolarizes, and increasing synaptic noise produces little effects on this consumption. That is, depolarizing spike threshold increases the energy utilization efficiency of the neuron in high firing rates. The lower values of energy consumption in one spike are achieved at more depolarized spike threshold and high stimulus current.

**Figure 8:**
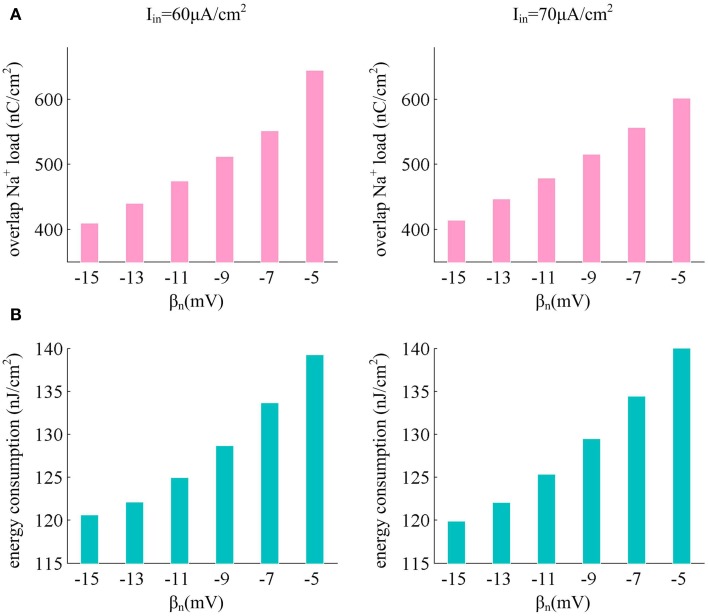
**Depolarizing spike threshold increases energy efficiency by reducing overlaps between Na^+^ and K^+^ currents. (A)** shows the overlap Na^+^ load for different spike threshold dynamics in the case of high stimulus. **(B)** gives the corresponding total energy required by a spike for each spike threshold dynamic. The stimulus current is *I*_*in*_ = 60μ A/cm^2^ and *I*_*in*_ = 70μ A/cm^2^, the noise amplitude is σ = 0μ A/cm^2^.

From the results in Figures 6B,C, it can be found that there are overlaps between Na^+^ and K^+^ currents in an action potential. These two positive charges flow in opposite directions as they pass through cell membrane, so that they can neutralize each other during the overlap. The overlap charge could be computed as the integral of Na^+^ current during the hyperpolarized phase of the spike (Moujahid et al., 2011, 2014; Moujahid and D'Anjou, 2012), which is the inward Na^+^ that is counterbalanced by outward K^+^. Previous studies (Alle et al., 2009; Carter and Bean, 2009; Sengupta et al., 2010, 2013; Moujahid and D'Anjou, 2012; Moujahid et al., 2014) have shown that reducing this overlap load could decrease the energy demands for spike generation. From Figure 8A, one can find that the overlap Na^+^ indeed undergoes a reduction as spike threshold gets depolarized in the case of high *I*_*in*_ values. The efficient use of inward Na^+^ could decrease the energy consumption in an action potential and enhance the energy efficiency of the neuron (Figure 8B).

## Discussion

Our results demonstrate there is a fundamental connection between spike threshold dynamics and neuronal input-output properties. When spike threshold is insensitive to *dV*/*dt*, the *f* − *I*_*in*_ curve is continuous and weak noise is unable to produce inhibitory effects on spiking rhythms. In this case, the neuron generates a type I PRC that exclusively displays phase advances. However, when spike threshold is sensitive to *dV*/*dt*, the neuron generates a discontinuous *f* − *I*_*in*_ curve and a type II PRC in the cases of no or low noise. Increasing noise amplitude switches the *f* − *I*_*in*_ curve from discontinuous to continuous. Simultaneously, weak synaptic noise obviously prohibits spiking rhythms when *I*_*in*_ is near and above the bifurcation point I^*^_in_. In this case, as the inverse relationship between spike threshold and *dV*/*dt* gets pronounced, the inhibitory effects of weak noise on spiking rhythms and the discontinuity of *f* − *I*_*in*_ curve both become more significant. Further, the depolarization of the spike threshold shifts the *f* − *I*_*in*_ curve to the right, alters the slope of *f* − *I*_*in*_ curve at low spike rates, and increases the current threshold for evoking neuronal repetitive spiking. These results indicate that the spike threshold properties, such as, whether it is sensitive to *dV*/*dt*, the inverse degree of it depends on *dV*/*dt*, or even the values of threshold potential could all obviously influence neuronal input-output relations.

All these input-output properties associated with each spike threshold dynamic are derived from the distinct nonlinear interactions between inward (depolarizing) and outward (hyperpolarizing) currents at the perithreshold potentials. When spike threshold is insensitive to *dV*/*dt*, the outward *I*_*K*_ does not activate prior to spike threshold, which leads inward *I*_*Na*_ to dominate spike initiation without the restraint of *I*_*K*_. Due to the absent of outward *I*_*K*_, the inward *I*_*Na*_ is able to balance weak outward currents at the perithreshold potentials, which results in a non-monotonic *I*_*SS*_ − *V* curve, a type I PRC, and a SNIC bifurcation. Under these conditions, *V* could be forced to slowly pass through threshold potential and the neuron is able to spike at low frequencies, thus producing a continuous *f* − *I*_*in*_ curve. Since the SNIC bifurcation does not have the bistable region, the inhibitory effects of weak noise on spiking rhythms is missing in this case. When spike threshold is sensitive to *dV*/*dt*, the outward *I*_*K*_ is able to activate at the subthresholds, and could become sufficiently strong prior to spike initiation. Then, inward *I*_*Na*_ is unable to balance it at the perithreshold potentials, which leads to a monotonic *I*_*SS*_ − *V* curve, a type II PRC and a Hopf bifurcation. The action potential could be successfully initiated because inward *I*_*Na*_ activates quickly to drive *V* through threshold with a sufficient speed that slow outward *I*_*K*_ cannot overtake. This means the neuron is unable to spike at low rates, which corresponds to a discontinuous *f* − *I*_*in*_ curve. Since the neuron generates a narrow bistable region when Hopf bifurcation occurs, the weak noise could convert its state from stable limit cycle to resting and then prohibit repetitive spiking. Further, the increase of current threshold for evoking repetitive spiking is also due to the intensity of net outward current becomes stronger as threshold gets depolarized.

The biophysical explanation about how the activation properties of intrinsic membrane currents contribute to the spike threshold dynamic with the preceding *dV*/*dt* has been reported in many experimental and modeling studies (Hodgkin and Huxley, 1952; Storm, 1988; Azouz and Gray, 2000, 2003; Bekkers and Delaney, 2001; Henze and Buzsáki, 2001; Dodson et al., 2002; Wilent and Contreras, 2005; Guan et al., 2007; Goldberg et al., 2008; Higgs and Spain, 2011; Wester and Contreras, 2013; Fontaine et al., 2014). Meanwhile, the biophysical basis of how different dynamical mechanisms of spike initiation (i.e., SNIC and Hopf bifurcation) generate distinct input-output relations, such as Hodgkin class 1 and class 2 excitability (Koch, 1999; Izhikevich, 2005; Prescott and Sejnowski, 2008; Prescott et al., 2008a,b; Yi et al., 2014a) or type I and type II PRC (Ermentrout, 1996; Smeal et al., 2010; Fink et al., 2011), has also been well established. However, none of them has explored how spike threshold dynamic modulates neuronal input-output relation. With a simple biophysical model, we have successfully identified a fundamental connection between spike threshold dynamic and input-output property in this study. We also provided a biophysical interpretation about how the nonlinear interactions between inward and outward currents at the perithersholds contribute to such connection. The powerful predictive ability of subthreshold biophysical properties is further attested in our work, which may be conducive to increase its future applications in neural coding.

Since the stochasticity is a prominent feature of neural system (Tuckwell, 1989; Gerstner and Kistler, 2002; Tuckwell and Jost, 2010), much effort has been devoted to exploring what effects of noise may produce on neuronal activity. A lot of modeling and experimental studies have reported that noise is able to enrich neuronal stochastic dynamics and trigger many complex behaviors near different bifurcation points. For example, it may induce stochastic firing patterns and enhance neuronal information transmission capability through coherence resonance near SNIC bifurcation (Gu et al., 2011; Jia et al., 2011; Jia and Gu, 2012), inhibit repetitive spiking through inverse stochastic resonance near Hopf bifurcation (Paydarfar et al., 2006; Tuckwell et al., 2009; Tuckwell and Jost, 2010, 2011, 2012; Guo, 2011), or completely destroy bifurcation scenarios and make neuronal response present a reliable feature (Tateno and Pakdaman, 2004). However, most of these studies focus on the phenomenological description of how noise impacts spiking behavior, while do not provide a satisfying explanation about the relation between neuronal intrinsic property and noisy effects. Unlike them, the present study associates noisy effects on spiking rhythms with neuronal intrinsic threshold dynamic. What is more, we provide a plausible biophysical interpretation for the observed noisy effects by relating them to the dynamical mechanism of spike initiation. All these investigations could provide a great insight into how noise participates in neural coding.

In addition, we adopt a novel approach proposed by Moujahid et al. (2011, 2014) and Moujahid and D'Anjou (2012) to characterize the electrochemical energy of the neuron with different spike threshold dynamics. This approach is based on the biophysical considerations about the nature of neuron model, which allows one to deduce an analytical expression of the electrochemical energy involved in the dynamics of the model. Contrary to the ion counting approach, this method does not need to calculate the number of Na^+^ required to depolarize membrane when estimating energy consumption, and also it requires no hypothesis about the extent of the overlapping between Na^+^ and K^+^ (Moujahid et al., 2011, 2014; Moujahid and D'Anjou, 2012). Thus, it could avoid the overestimate value of energy that results from the ionic-counting based method (Attwell and Laughlin, 2001; Alle et al., 2009; Hertz et al., 2013). With this approach, we have found a basic link between spike threshold, energy efficiency, and spiking frequency. It is shown that the average energy consumption rate increases with spiking frequency and could detect the transition of the neuron from quiescence to firing state, whereas the energy demand of a single spike decreases with spiking frequency. This relation between energy consumption and spiking frequency is consistent with that observed in the neocortex, hippocampus, thalamus, and squid axon (Moujahid and D'Anjou, 2012; Moujahid et al., 2014). As spike threshold gets depolarized, the average energy consumption rate gets smaller. Meanwhile, the energy demand for generating an action potential in the case of high stimulus also decreases. This demonstrates that depolarizing spike threshold could increase the energy efficiency of the neuron. We further show that the more efficient use of electrochemical energy in the case of more depolarized threshold is mainly due to the reduced overlap load between inward Na^+^ and outward K^+^ currents. Previous reports (Alle et al., 2009; Carter and Bean, 2009; Sengupta et al., 2010, 2013; Moujahid and D'Anjou, 2012; Moujahid et al., 2014) have proposed that if the Na^+^ and K^+^ currents have the substantially reduced overlap, the corresponding action potential is more energy efficient. Our stimulation results are consistent with this proposal. All these experimental and modeling observations suggest that the interactions between inward and outward currents could also determine the electrochemical energy required by the neuron to generate action potentials.

## Conclusion

A dynamic spike threshold dependent on *dV*/*dt* plays a vital role in neural coding and spike initiation, which requires a number of metabolic energy. In this work, we have used a modified Morris-Lecar model to systematically investigate the input-output property and energy efficiency of the neuron with different spike threshold dynamics. To the best of our knowledge, this is the first study that links spike threshold dynamics, biophysical properties, spike initiation, input-output relations and energy efficiency together. The predictions and relevant mechanistic explanations could be tested by intracellular recording *in vivo*, and simultaneously more biophysically realistic simulations will be required if we want to replicate these biological effects more accurately. The systematic investigation about how spike threshold dynamics modulates neural input-output properties and energy efficiency is a useful stepwise method for exploring how spike threshold participates in neural coding. Moreover, translating the phenomenological descriptions into biophysical interpretation is crucial for revealing how membrane biophysics impacts neural coding. Thus, our stimulations could contribute to uncover the functional significance of spike threshold as well as biophysical properties in neural coding mechanism.

### Conflict of interest statement

The authors declare that the research was conducted in the absence of any commercial or financial relationships that could be construed as a potential conflict of interest.
